# Supplementary material for: The Evolution and Characterization of the RNA Interference Pathways in Lophotrochozoa
Source: Genome Biol Evol. 2024 May 7;16(5):evae098. doi: 10.1093/gbe/evae098 (PMC11114477; doi:10.1093/gbe/evae098)
Supplement: evae098_Supplementary_Data [file evae098_supplementary_data.zip › Supplementary_materials.zip!/selected_miRNAs/NoGe_selected_miRNAs/novel_miRNA_families_pdf_files/NMRB01000697.1_Notospermus_geniculatus_strain_Ushimado_scaffold697,_whole_genome_shotgun_sequence_18912.pdf]

Provisional ID	:	NMRB01000697.1_Notospermus_geniculatus_strain_Ushimado_scaffold697,_whole_genome_shotgun_sequence_18912
Score total	:	49.3
Score for star read(s)	:	3.9
Score for read counts	:	44.5
Score for mfe	:	1.5
Score for randfold	:	0
Score for cons. seed	:	-0.6
Total read count	:	99
Mature read count	:	59
Loop read count	:	0
Star read count	:	40

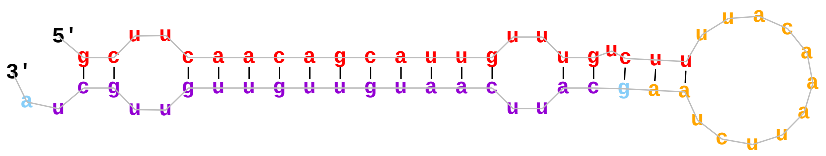

Genome coordinates: NMRB01000697.1 40338-40395

pre-miRNA sequence:
GCTTCAACAGCATTGTTTGTCTTGCTTCAACAGCATTGTTTGTCTTTTACAAATTCTAAGCATTCAATGTTGTTGTTGCT

Mature sequence: GCTTCAACAGCATTGTTTGTCTT

BLAST results for the pre-miRNA sequence against the *Lineus longissimus* genome assembly:

Scaffold	Start	Stop	Identity	e-value
OU343000.1 1	10291937	10291921	100.000	2.1

```
Nge_novel_miRNA          GCTTCAACAGCATTGTT
Llo_genome               GCTTCAACAGCATTGTT
```
